# Minimally Invasive Surgical Strategies in Intraventricular Tumors: Preliminary Experience with Tubular Retractors for a Personalized Approach in Intraventricular Meningiomas

**DOI:** 10.3390/jpm16020061

**Published:** 2026-01-27

**Authors:** Alessio Iacoangeli, Valentina Liverotti, Mario Chiapponi, Denis Aiudi, Andrea Mattioli, Lucia di Somma, Andrea Carai, Michele Luzi, Roberto Trignani, Hani A. Mahboob, Gustavo Luzardo, Alberto Feletti, Carlo Efisio Marras, Maurizio Iacoangeli, Maurizio Gladi

**Affiliations:** 1 Neurosurgery Clinic of Azienda Ospedaliero Universitaria delle Marche, Marche Polytechnic University, 60126 Ancona, Italy; valentina.liverotti@ospedaliriuniti.marche.it (V.L.); mario.chiapponi.med@gmail.com (M.C.); denis.aiudi@gmail.com (D.A.); andrea.mattioli.92@gmail.com (A.M.); luciagiovannamaria.disomma@ospedaliriuniti.marche.it (L.d.S.); neurotra@gmail.com (M.I.); maurizio.gladi@ospedaliriuniti.marche.it (M.G.); 2 Neurosurgery Division of Azienda Ospedaliero Universitaria delle Marche, 60126 Ancona, Italy; michele.luzi@ospedaliriuniti.marche.it (M.L.); roberto.trignani@ospedaliriuniti.marche.it (R.T.); 3 Neurosurgery Unit, Department of Neurosciences, Bambino Gesù Children’s Hospital, IRCCS, 00165 Rome, Italy; andrea.carai@opbg.net; 4 Department of Neurological Surgery, King Fahad General Hospital Ministry of Health, Jeddah 21442, Saudi Arabia; hmahboob@moh.gov.sa; 5 Department of Neurosurgery, University of Mississippi Medical Center, Jackson, MS 2500, USA; gluzardo@umc.edu; 6 Department of Neurosciences, Biomedicine, and Movement Sciences, Institute of Neurosurgery, University of Verona, 37126 Verona, Italy; alberto.feletti@univr.it; 7 Department of Neurosurgery, Hospital del Mar, 08003 Barcelona, Spain; carlefis64@gmail.com; 8 Systems Neurology and Neurotherapeutics Lab Neurosciences Program, Hospital del Mar Research Institute, 08003 Barcelona, Spain

**Keywords:** meningioma, minimally invasive, tubular retractor, port technique, intraventricular, tumor, elderly, personalized medicine

## Abstract

**Background**: Intraventricular tumors represent a minority in the context of brain tumors, but their surgical treatment is particularly complex due to their vascularization and visualization, especially in deep localization. The characteristics of these tumors make them ideal candidates for minimally invasive surgical strategies such as the tubular retractor technique, above all in the elderly population. **Objectives**: A 1-year multi-center, retrospective case series was performed: the authors describe their preliminary experience using a neuronavigated tubular retractor in the management of 11 cases of intraventricular meningiomas. **Methods**: Clinical and radiological findings were examined to define the outcomes. We used an alternative tubular retractor system obtained using a modified preexisting general surgery trocar (ENDOPATH XCEL 15 mm trocar) or the NICO System BrainPath. **Results**: Gross total resection, defined as the removal of all the tumor visible from the brain scans, was achieved in all cases. Ten out of eleven of the patients did not experience major complications or permanent neurological deficits. Four patients presented transitory post-operative agitation, visual blurring and transient hemiparesis. All patients (mean age 72.6 years) were discharged from the hospital in 5–7 days. **Conclusions**: Our preliminary experience suggests that the use of navigated tubular retractors, by displacing the fibers and hence minimizing the damage to the surrounding cerebral parenchyma, is feasible and safe, representing a minimally invasive technique for a personalized and patient-tailored approach. The use of the selective ultrasonic aspirator makes it possible to excise the tumor through the narrow corridor of the tubular lumen of around 2 cm, and this technique can also be improved using both endoscope and microscope guidance.

## 1. Introduction

Intraventricular tumors represent a minority in the field of brain neoplasms, especially meningiomas (less than 4% of all meningiomas on average) [1], but their treatment is particularly complex due to the fine vascularization and their deep location. In clinical practice there has been an evolution towards minimally invasive techniques with the aim of preserving the neurological function; an example of this concept is the technique proposed by Yasargil regarding the use of trans-cisternal trajectories rather than the usual transcortical surgical corridors [2]. The characteristics of these tumors, when located deep in the brain, make them ideal candidates for resection using a tubular retractor. This procedure grants direct access to the tumoral mass and the possibility to minimize the injury to the surrounding brain tissue [3]. In addition, it represents a versatile technique that adapts to both the microscopic and the endoscopic instrumentation. Intraventricular meningiomas originate from the choroid plexus’ stroma: arachnoid cells here are found to be secondary to the embryologic origin of the choroid plexus [4]. Jamshidi et al. and Lin et al. published the first reported series of intraventricular meningiomas and to date, to the best of our knowledge, most of the literature regarding the use of tubular retractors in intraventricular tumors is still based on individual case reports [1,2,3,4,5]. Beyond technical refinement, surgery for intraventricular meningiomas exemplifies personalized medicine in neurosurgery, where outcomes depend on tailoring the operative corridor to patient-specific ventricular anatomy, sulcal/gyral patterns, and the lesion’s relationship to functional networks. Modern planning tools (neuronavigation, tractography, and multimodal visualization) enable an individualized “precision” transcortical route designed to reduce collateral injury while preserving function, which is particularly relevant in older patients and in dominant-hemisphere lesions. In this context, tubular retractors can be viewed not only as a minimally invasive access tool, but also as an enabler of individualized parafascicular corridors that operationalize maximal safe resection on a case-by-case basis.

The aim of this study is to describe the surgical technique and present our experience in 11 cases of intraventricular meningiomas managed through the use of tubular retractors; our multicenter experience focuses on the practical, single patient-tailored and personalized use of an available trocar-based tubular system and a hybrid microscope–endoscope workflow for intraventricular meningiomas.

## 2. Materials and Methods

**Figure 1:**
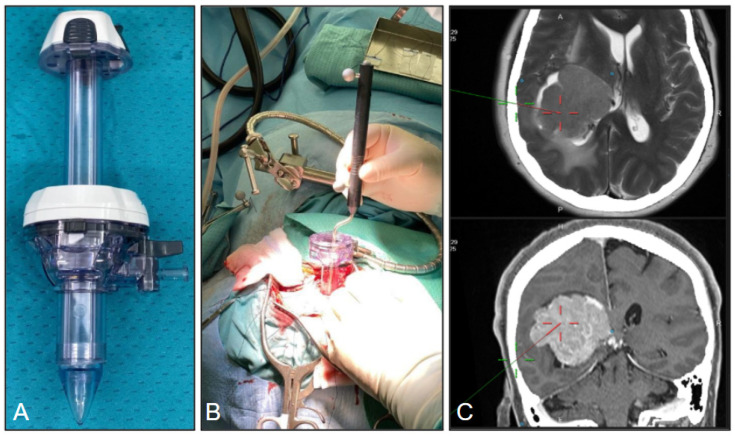
(**A**) The ENDOPATH XCEL 15 mm trocar tubular retractor. (**B**) Neuroavigator probe placed inside the port (secured to a Greenberg flexible arm), during retractor insertion under image guidance. (**C**) Intraoperative navigation images showing targeting of the lesion.

A retrospective 1-year multi-institution study (from January 2023 to December 2023) was conducted, including a series of 11 patients with intraventricular meningioma operated on using the tubular retractor. Surgical resection was performed with the assistance of the neuronavigation system, microscope, neuroendoscope, selective ultrasonic aspirator and tubular retractors. EVD was not routinely used. Surgical resection was assisted with a Leica surgical microscope (Leica Microsystems, Wetzlar, Germany) and a Stryker (Kalamazoo, MI, USA) rigid endoscope with a 0-degree optic × 2 mm in diameter × 12 cm in length. The tubular retractor system was obtained from general surgery equipment and secured to the skull clamp with a Greenberg flexible arm, ENDOPATH XCEL 15 mm trocar (Ethicon, Johnson & Johnson MedTech, Somerville and Bridgewater, NJ, USA) (Figure 1) or, in a minority of cases (n = 3) with the NICO System BrainPath (NICO Corporation, Indianapolis, IN, USA).

We describe the technique regarding the excision of the tumoral mass using the tubular retractor. Patients’ demographics, onset and follow-up, clinical data (symptoms and neurological status), operative and postoperative details, histopathological diagnosis, and radiological imaging, including a study of the depth of the tumor, were analyzed to define outcome.

### 2.1. Perioperative Management

**Figure 2:**
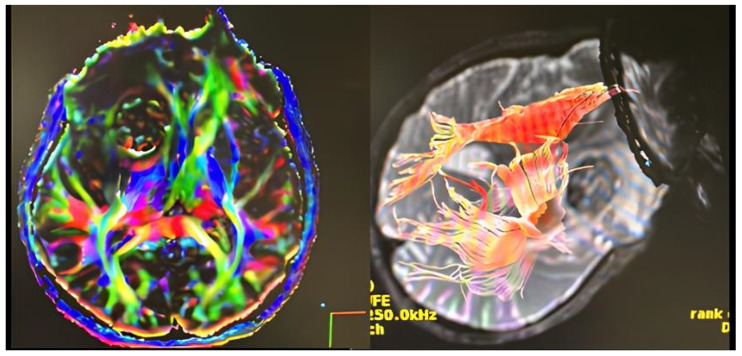
Representative preoperative diffusion tractography used for corridor planning in a dominant-hemisphere right intraventricular meningioma. Tractography reconstructions illustrating adjacent major subcortical pathways (e.g., optic radiations and language-related association fibers), integrated into neuronavigation to define a trans-sulcal parafascicular trajectory that avoids critical tracts.

All patients underwent magnetic resonance imaging with and without contrast, in the majority of cases (n = 8) supplemented by tractography along with the neuronavigation protocol, to determine the surgical trajectory and detailed tumor position (BrainLAB neuronavigation system, Munich, Germany). Measurements of the tumor, as well as its depth from the entry point on the cortical surface, were made on contrast-enhanced T1-weighted MRI sequences. The anatomic location of the tumor and its dimensions were determined in order to choose a patient-tailored surgical trajectory. An immediate CT scan and a follow-up MRI were carried out after 3 months and 1 year from surgery. Surgical corridors were selected using contrast-enhanced MRI integrated into neuronavigation and tailored to tumor location, depth, ventricular anatomy, and patient-specific sulcal/gyral landmarks. When available, diffusion-based tractography was used to identify and avoid major subcortical pathways relevant to the chosen hemisphere and corridor (e.g., optic radiations for atrial/trigonal lesions; arcuate fasciculus/superior longitudinal fasciculus for dominant hemisphere trajectories; corticospinal tract for more anterior or superior corridors). The entry point was preferentially trans-sulcal when feasible; otherwise, a limited corticectomy (15–20 mm) was planned over a non-eloquent gyrus aligned with the long axis of the intended corridor. Formal awake mapping and direct cortical stimulation were not routinely employed in this retrospective cohort; this limitation is addressed in the Discussion. A representative example of tractography-based planning for a dominant-hemisphere right ventricular lesion is shown in Figure 2.

### 2.2. Operative Technique

**Figure 3:**
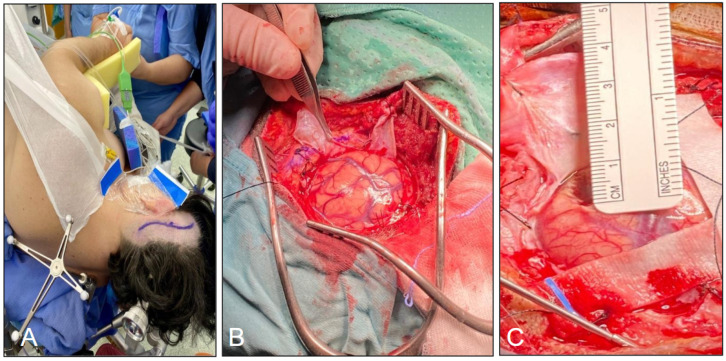
(**A**) The lazy S incision in the lateral position. (**B**) Example of durotomy performed in an x-shaped manner. (**C**) Round 3–4 cm craniotomy and corticectomy no larger than 15–20 mm.

After general anesthesia, we adopted for the patients a supine, lateral or prone position according to the location of the tumor and the tailored approach. The Mayfield (Ohio Medical Instrument Co., Ltd., Cincinnati, OH, USA) skull clamp was used and a surface neuronavigation registration was performed with the BrainLAB system. A linear or lazy S incision, in association with a round 3–4 cm craniotomy, was planned in all cases. Durotomy was preferably performed in an x-shaped fashion or in a semilunar manner (Figure 3).

**Figure 4:**
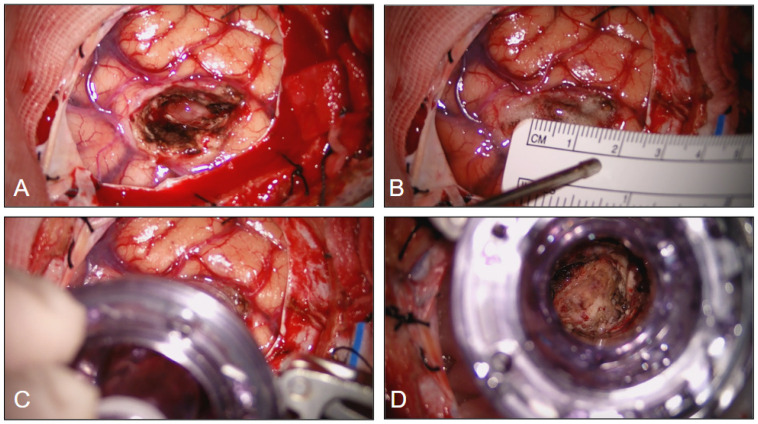
(**A**,**B**) A 15–20 mm corticectomy before positioning of the tubular retractor system. (**C**,**D**) A 25 mm trocar placed through the corticectomy with gentle retraction and intraoperative view through its lumen.

**Figure 5:**
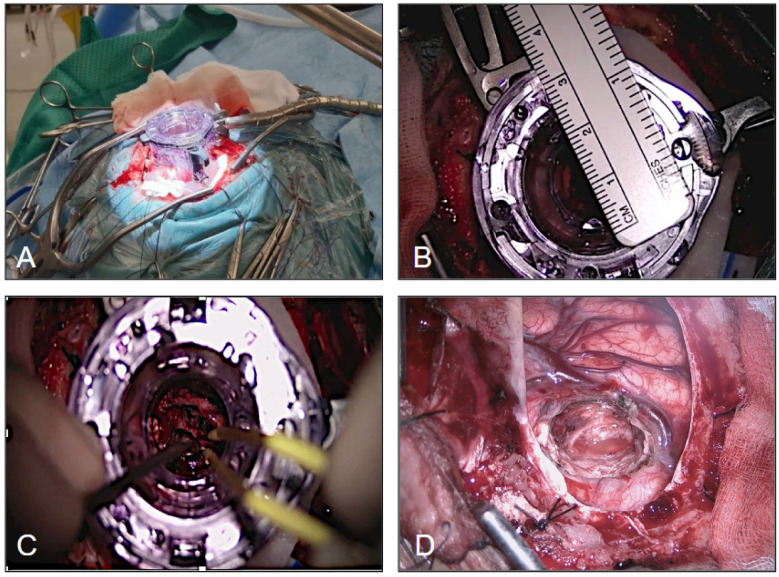
(**A**) Trocar tubular retractor system positioning. (**B**) Top view of the working lumen. (**C**) Simultaneous use of bipolar forceps and suction through the trocar. (**D**) View of the surgical field after tubular retractor removal.

A transcortical approach was used in our series and, after identifying the reference gyrus and sulci, a trans-sulcal approach or corticectomy no larger than 15–20 mm was performed. The trocar tubular retractor was introduced (the working lumen on top, once the blunt introducer was removed, was about 20 mm), perpendicular to the cortical surface, and inserted gently and in rotation into the brain tissue, always guided by the neuronavigation probe inside the port. These maneuvers bluntly spread the white matter fibers, permitted dynamic retraction and minimized brain tissue disruption, avoiding high and prolonged brain retraction pressure as an effect of the 360-degree force distribution. Once the tumor was reached, the blunt introducer, used for the blunt dissection of the white matter tracts, was removed, creating the direct-angle working space. The working angle can eventually be modified during surgery; to explore the operative bed and reduce the risk of residual tumor, we favored endoscopic inspection through the fixed port and minor, navigation-guided adjustments in angulation rather than wide “sweeping” movements. Any change in trajectory was performed under direct visualization with the port secured to a flexible arm to minimize torque on the surrounding parenchyma. Hemostasis was obtained with standard microsurgical technique (bipolar coagulation, irrigation, and hemostatic adjuncts as needed) while maintaining ventricular irrigation to limit ventricular collapse. A Greenberg flexible arm was then secured to the skull clamp and attached to the trocar system (Figure 4 and Figure 5).

The choice of port diameter is influenced by the diameter of the lesion and by the preoperative radiological measurements. We performed tumor resection by means of microscope and endoscope. The tumor was debulked using the ultrasonic selective aspirator and angulation of the tubular retractor in multiple directions. Once tumor resection and hemostasis were achieved, the tubular retractor was slowly removed, in a rotational way, as when it was inserted. In none of the cases was an EVD (external ventricular drain) placed routinely before the procedure (Figure 5). The study was conducted in accordance with the Declaration of Helsinki and ethical approval was waived by our local ethics committee in view of the retrospective nature of the study and the procedures performed being part of routine care.

## 3. Results

### 3.1. Patient Overview

During 2023, 11 patients (5 males and 6 female), with an overall mean age of 72.6 years (range 56–83), were operated for an intraventricular meningioma by means of the tubular retractor system. In four patients the tumor was located in the trigonal area of the right lateral ventricle, in five patients in the left trigonal area of the ventricle, and in two patients in the region of the foramen of Monro. In all patients the resection was gross total. In no case, before or after the operation, was it necessary to use an EVD. None of the patients, except one patient with postoperative right-side hemiplegia probably caused by venous infarction, experienced permanent neurological deficits. Four patients presented transitory post-operative agitation, visual blurring and transient hemiparesis. There were less than 10% permanent major complications related to surgery (one patient with postoperative right-side hemiplegia probably caused by venous infarction) and no mortalities. Surgical procedures were performed with microsurgical vision and neuroendoscopic assistance in all cases. Mean tumor depth was 1.72 cm and average preoperative tumor volume for the series was 45.1 cm^3^; tumor types included N = 9 WHO grade I and N = 2 grade II meningiomas (Table 1 and Table 2). Diffusion-based tractography for corridor planning was available in most cases (Table 3); given the retrospective design, tractography acquisition and the selection of specific tracts of interest were not standardized across centers.

### 3.2. Illustrative Cases

**Figure 6:**
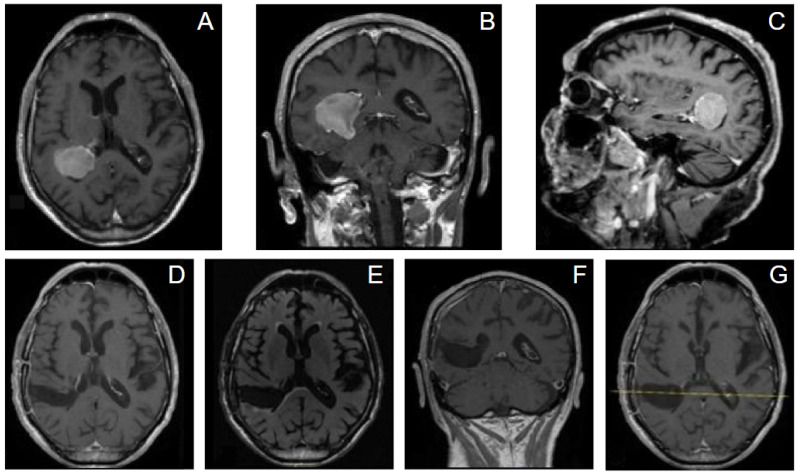
(**A**–**C**) The axial (**A**), coronal (**B**) and sagittal (**C**) contrast-enhanced, preoperative MRI showed a heterogeneously enhancing mass arising from the trigone of the right lateral ventricle. (**D**,**E**) The axial contrast enhanced postoperative MRI (**D**) revealed a complete tumor resection with minimal FLAIR changes (**E**) demonstrating no damage to the brain immediate surrounding the tubular trajectory. (**F**,**G**) At 3 months from surgery there is no evidence of tumor recurrence and/or evidence of brain injury.

**Figure 7:**
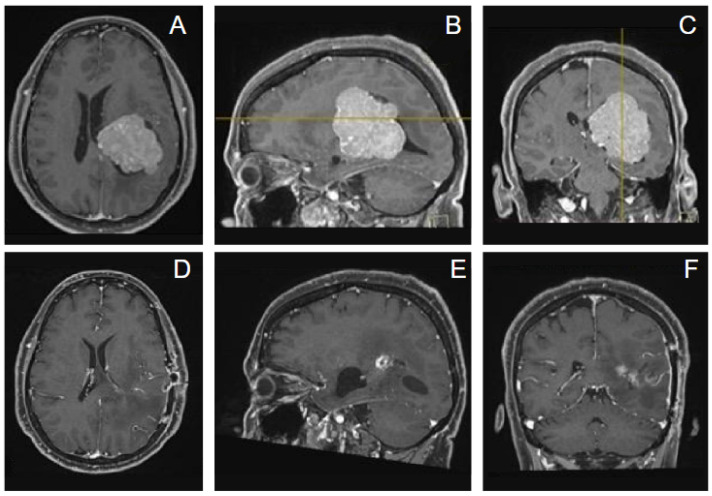
The axial (**A**), sagittal (**B**), and coronal (**C**) contrast-enhanced preoperative MRI showed a large intraventricular mass in the left trigone with peritrigonal extension. (**D**–**F**). Contrast-enhanced postoperative MRI revealed a complete tumor resection, with minimal brain damage.

**Figure 8:**
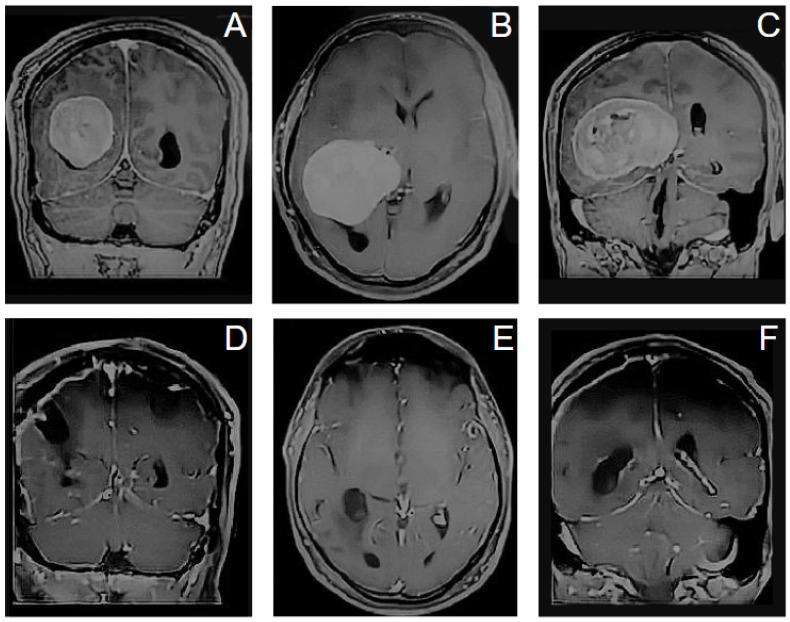
Preoperative (first row (**A**–**C**)) and postoperative (second row (**D**–**F**)) MRI images showing removal of a large homogeneously enhancing tumor involving the right ventricular trigone that appeared consistent with a meningioma.

- Case 1. An 83-year-old man presented with a history of loss of consciousness episodes and ideomotor slowdown. He was otherwise neurologically intact. MRI revealed a large right-sided intraventricular mass in the trigone of the right lateral ventricle consistent with meningioma. We adopted for the patient a supine position, and a minimally invasive tumor excision was performed using the tubular retractor system assisted by neuronavigation. Gross total resection (GTR) was achieved and pathology confirmed WHO grade 1 meningioma. He had an uneventful recovery and remained neurologically intact after surgery. At 3 months from surgery there was no evidence of tumor recurrence, and no evidence of brain injury or progressive neurological deficits (Figure 6).

- Case 2. A 56-year-old woman presented with memory impairment; dysarthria and neurological exam revealed hyposthenia at the right limbs. A contrast-enhanced MRI showed a large intraventricular mass in the left trigone with peritrigonal extension consistent with meningioma (Figure 7). We adopted for the patient a lateral position and a trans-sulcal temporal approach with port technique, neuronavigation-assisted. Surgery was uneventful and a gross total resection was obtained. Pathology revealed a WHO grade 2 meningioma. Postoperative MRI confirmed complete resection of the tumor. Adjuvant radiation therapy was not recommended due to complete tumor resection; the patient was neurologically intact and followed up.

- Case 3. A 79-year-old man presented with memory impairment, ideomotor slowdown and left hemitemporal hemianopsia; he was otherwise neurologically intact. Routine magnetic resonance imaging (MRI) revealed a large homogeneously enhancing tumor involving the right ventricular trigone that appeared consistent with a meningioma. He was scheduled for operation, and we adopted for the patient a prone position. Resection was achieved via the posterior aspect of the superior parietal lobule with a neuronavigated port technique. Surgery was uneventful and a total resection was obtained. Pathology revealed a WHO grade I meningioma. He recovered uneventfully and, at 1-year postoperative follow-up, there was no evidence of tumor recurrence, and no evidence of brain injury or progressive neurological deficits (Figure 8).

## 4. Discussion

In cerebral intraparenchymal tumors, brain injury related to retraction is a frequent complication with the onset of postoperative seizures, neurological deficits, brain edema and bad clinical outcomes [5,6,7]. Minimally invasive techniques, in transcortical approaches, make it possible to reduce damage to the surrounding brain, maintaining good visualization for these deep-located tumors. In this light, the use of circular or tubular retractors, known since 1988 [8], meets these purposes by dissipating the amount of retraction force in a circumferential manner, reducing the cortex under retraction’s amount of force per unit [5,8,9]. In intraparenchymal and intraventricular tumor surgery, retraction-related injury remains a relevant source of morbidity, including seizures, edema, contusion, and neurological deficits. Minimally invasive transcortical strategies aim to reduce cortical transgression and subcortical manipulation while maintaining adequate visualization for safe resection. Tubular retractors, originally introduced for stereotactic microsurgery, distribute forces circumferentially and can create a parafascicular working corridor that may lessen focal cortical pressure compared with fixed blade retractors. Several groups have advocated “retractorless” microsurgery and dynamic retraction, using instruments and gravity to minimize fixed pressure on the cortex during deep approaches. Conceptually, a tubular retractor corridor shares the same goal—limiting sustained focal pressure—yet differs in that it provides a stable, circumferential working channel. In our practice, the port was combined with trans-sulcal entry when feasible and with microinstruments plus endoscopic assistance to optimize visualization within a narrow corridor. These approaches should be viewed as complementary rather than competing: dynamic retraction may be preferred when a wider working angle is required, whereas a tubular corridor may be advantageous when a small corticectomy and a stable route to a deep ventricular target are priorities. The circumferential shape of the tubular port theoretically redistributes pressure equally across the surrounding tissue in a 360◦ dispersion pattern, and produces less direct cutting and tearing trauma to the underlying brain tissue [10,11,12,13]. In our preliminary experience and in accordance with the literature, it was a safe technique, but there are authors who, in order to avoid potential complications such as shear injuries, have proposed alternative techniques, which are matter of further investigation [14,15,16].

The literature reports the use of different types of tubular retractor systems commercially available: as previously reviewed by Okasha et al., the most frequently mentioned tubular retractor system in the current literature is the ViewSite Brain Access System manufactured by Vycor Medical (Florida, United States) [5]; other authors used the BrainPath (BrainPath NICO Corporation, Indianapolis, Indiana, USA) [17], proposed a new mini tubular port like Liu et al. [10], or modified preexisting equipment like syringes and a spinal tubular retractor [9,18]. In our case, except for a few cases where the BrainPath was available, we modified a preexisting general surgery trocar as a tubular brain retractor. We used the ENDOPATH XCEL 15 mm trocar: this laparoscopic instrument is widespread and well described in the context of general surgery and, according to the literature, is a safe guide that potentially guarantees a low insertion force [19]. The technical features of the trocar seem to have met the expectations of reducing tearing trauma to the brain surrounding the lesion, due to the 360◦ pression dispersion pattern. Further studies to confirm our preliminary results are needed. Immediate postoperative CT was obtained in all patients primarily to screen for hemorrhage and corridor-related contusion/edema. However, edema along the corridor was not prospectively quantified in this retrospective series, limiting direct comparison with “no-retractor” series or conventional transcortical exposures. Prior quantitative imaging studies in other deep lesion cohorts have suggested that tubular retractors can reduce postoperative FLAIR signal change compared with traditional retraction strategies, supporting the rationale for this approach; future work should include standardized volumetric edema assessment in intraventricular meningiomas specifically. While prior reports have described port-based strategies for intraventricular lesions—often as case reports or small series—our multicenter experience focuses on the practical use of an available trocar-based tubular system and a hybrid microscope–endoscope workflow for intraventricular meningiomas, with gross total resection in all cases and predominantly transient morbidity. The approach is reproducible because it relies on standard neuronavigation, a small craniotomy, limited corticectomy, and commonly available visualization and debulking tools.

Deep-seated tumors could benefit from a minimally invasive approach with tubular retractors because they provide a small corticectomy and direct path to the lesion; this technique minimizes brain injury without adhering to the brain [3]. The transparency of most of the retractors offers a detailed visualization of surrounding tissue and the technique is versatile, using microscope, endoscope and routine instruments (such as the ultrasonic aspirator) as needed. Often, intraventricular tumors represent a challenge due to the poor visualization of the surgical field: tubular retractors, in addition to less invasive corticectomy and stability on surgical corridors, allow the use of both hands of the surgeon to manage other instruments when needed (microscope, exoscope, ultrasonic aspirator). Selecting the surgical corridor is the critical decision point for ventricular surgery. We tailored trajectories to lesion location, depth, ventricular anatomy, and sulcal/gyral landmarks, and we used diffusion-based tractography in most cases to identify and avoid major white matter pathways relevant to the planned hemisphere and corridor (particularly optic radiations for atrial lesions and language-related association fibers for dominant hemisphere approaches). Nonetheless, tractography has known limitations and does not replace functional mapping. In this retrospective cohort, formal awake mapping and direct cortical stimulation were not routinely used, including for left-sided lesions; this should be considered a limitation. Recent work emphasizes the value of integrating preoperative mapping and intraoperative neuromonitoring with parafascicular tubular approaches, and awake parafascicular strategies have been described for lesions near language networks; these evolving paradigms may further reduce postoperative deficits and merit prospective evaluation [20,21,22,23,24,25]. A frequent concern with tubular corridors is whether angulation risks shear injury when searching for residual tumor. In our technique, we preferentially used endoscopic inspection through the fixed port and performed only minor, navigation-guided adjustments rather than wide sweeping movements. The port was rigidly secured to reduce torque, and inspection/hemostasis were completed under direct visualization. We did not observe injuries clearly attributable to port manipulation; however, systematic diffusion-weighted imaging or connectome-based outcome measures were not incorporated and should be considered in future studies. Two tumors were WHO grade II. Given the small number and limited follow-up of this preliminary series, we cannot draw conclusions regarding comparative recurrence risk or the role of adjuvant therapy. In general, histological grade informs follow-up intensity and multidisciplinary discussion; longer follow-up and larger cohorts are needed to define outcomes for grade II tumors treated via port-based intraventricular approaches.

Our findings support framing intraventricular meningioma resection as a precision, personalized neurosurgery, in which the key determinant is not only tumor resectability but also the patient-specific relationship between the ventricular lesion and surrounding structural and functional pathways. In our workflow, corridor selection was individualized using MRI integrated into neuronavigation and, when available, diffusion tractography to identify pathways at risk and support planned avoidance. Within this paradigm, the tubular retractor functions as a technical enabler of personalization: it provides a stable parafascicular channel aligned to the chosen trajectory, permitting bimanual microsurgery and endoscopic inspection through a small corticectomy while aiming to minimize retraction-related injury.

From a personalized-medicine perspective, future work should move from technique description to patient-stratified decision frameworks that incorporate (i) tumor location/ventricular anatomy and depth to target, (ii) dominant-hemisphere considerations and language lateralization, (iii) tractography/connectome-informed risk mapping, and (iv) individualized postoperative surveillance guided by histological grade and recurrence risk. Prospective studies should also include standardized quantification of corridor-related imaging changes (e.g., edema/contusion) and patient-centered functional outcomes to better define which anatomic and clinical profiles derive the greatest benefit from port-based intraventricular surgery.

The need for a minimally invasive technique, aimed at improving outcome and reducing complications, is particularly necessary in elderly patients and reflects a modern concept of neurosurgery: our series included a population with an average age of 72.6 years. In our experience, according to the literature, older age did not seem to be a limit and promising technologies such as tractography or Extend Reality could additionally help [26,27,28,29,30,31].

We believe in and prefer, in accordance with the literature [3], using both microsurgical technique and endoscopic assistance in the management of intraventricular tumors. The micro-endoscopic approach, in our preliminary experience, seemed to be the fastest approach related to the greatest possibility of a complete deep tumor resection. The purpose of this study is to describe our preliminary experience with intraventricular meningiomas approached through tubular retractors. As documented in recent studies in the literature, our, even if preliminary, experience seems to confirm that this technique is safe and allows the total removal of intraventricular meningioma.

Pearls and pitfalls (practical points).

1. Plan the corridor first: prioritize a trans-sulcal parafascicular route aligned to the ventricular target, integrating tractography when available.
2. Keep the corticectomy small (15–20 mm) and avoid unnecessary cortical coagulation.
3. Secure the port to minimize torque; favor endoscopic inspection over wide angulation.
4. Control CSF egress with irrigation to reduce ventricular collapse and maintain visualization.
5. Have a conversion strategy: if uncontrolled bleeding or inadequate working angle occurs, broaden exposure early rather than persist with unsafe constraints.

Limitations: this is a retrospective, small series without a control group and without standardized, quantitative measures of corridor-related edema or network-level outcomes. Tractography and mapping strategies were not uniform across centers, and awake mapping/direct cortical stimulation were not routinely used. These limitations underscore the need for prospective studies incorporating standardized imaging and functional outcome metrics.

## 5. Conclusions

Our preliminary experience suggests that the use of tubular retractors in intraventricular neoplastic diseases such as meningiomas is versatile, feasible and safe. The use of this minimally invasive technique allows for the reduction of damage to the cerebral parenchyma by displacing the fibers surrounding the tubular retractor in a 360-degree manner. It also made it possible to work, easily, with all the main ordinary surgical instrumentation, as well as selective ultrasonic aspirator, microscope and endoscope, through its narrow lumen of a few centimeters; this strategy aligns with the principles of personalized medicine in neurosurgery where the operative corridor can be individualized using patient-specific imaging and, when available, tractography to mitigate functional risk and preserve neurological outcomes in deep ventricular targets.

## Figures and Tables

**Table 1:** Patient overview. M: male, F: female, y: years, WHO: World Health Organization, GTR: gross total removal, i.e., removal of all of the tumor visible from the brain scans, R: right, L: left.

Data	Description
Number of patients	11
Age (years)	mean 72.6; range 56–83
Sex	M: 5; F: 6
Location	Trigone/atrium: R 4, L 5; Foramen of Monro region: 2
Histology	WHO grade I: 9; WHO grade II: 2
Mean pre-op tumor volume (cm^3^)	45.1
Mean tumor depth (cm)	3.72
Extent of resection	GTR in 11/11
Permanent major deficit	1/11 (venous infarction suspected)
Transient postoperative symptoms/deficits	4/11

**Table 2:** Patient detailed overview: surgical corridors and tracts at risk are exposed.

Pt	Tumor Region	Corridor/Entry Gyrus (Trans-Sulcal when Possible)	Tracts at Risk
1	Right trigone/atrium	Posterior superior parietal lobule via intraparietal sulcus (IPS) → atrium	Optic radiations (posterior/Baum’s loop); SLF (parietal segment); callosal fibers near splenium; cingulum (posterior)
2	Right trigone/atrium	Precuneus via parieto-occipital sulcus → medial atrium	Optic radiations; forceps major/splenial fibers; posterior cingulum; inferior fronto-occipital fasciculus (IFOF, posterior)
3	Right trigone/atrium	Superior occipital gyrus	Optic radiations (high risk); forceps major; IFOF (posterior); inferior longitudinal fasciculus (ILF)
4	Right trigone/atrium	Angular gyrus region via posterior IPS	SLF; IFOF; posterior optic radiations; parietal “attention” networks (non-dominant but clinically relevant)
5	Left trigone/atrium	Posterior superior parietal lobule via IPS → atrium	Optic radiations; SLF/arcuate complex (dominant); IFOF (dominant semantic pathway); splenial/callosal fibers
6	Left trigone/atrium	Precuneus via parieto-occipital sulcus → medial atrium	Optic radiations; forceps major/splenium; posterior cingulum; IFOF (posterior)
7	Left trigone/atrium	Posterior middle temporal gyrus	Meyer’s loop (optic radiations, very high risk); ILF; IFOF; arcuate/SLF (dominant)
8	Left trigone/atrium	Inferior parietal lobule (supramarginal/angular region)	Arcuate fasciculus/SLF (language); IFOF; optic radiations; parietal language cortex (dominant)
9	Left trigone/atrium	Superior parietal lobule	SLF/arcuate; optic radiations (posterior); cingulum; callosal fibers (posterior)
10	Foramen of Monro region	Middle frontal gyrus via superior frontal sulcus/frontal horn (transcortical transventricular to Monro)	Corticospinal tract (subcortical); SLF (frontal segment); frontal aslant tract (speech initiation, dominant); anterior thalamic radiation; caudate head
11	Foramen of Monro region	Superior frontal gyrus (more medial frontal horn trajectory; minimized eloquent lateral cortex)	Cingulum; forceps minor (callosal frontal fibers); anterior thalamic radiation; corticospinal tract (if too posterior); fornix (critical near Monro, memory)

**Table 3:** Tractography use, retractor type and specific postoperative deficit.

Pt	Tumor Region	Case-Specific Complication	Case Detail (Likely Substrate, Tractography Use and Retractor Type)
2	Right trigone/atrium	Transient homonymous quadrantanopia improving over weeks	Optic radiations stretch; no tractography; NICO retractor
4	Right trigone/atrium	Transient left visuospatial neglect (or mild inattention), resolved by discharge/short rehab	Non-dominant parietal network corridor edema; tractography performed; ENDOPATH retractor
6	Left trigone/atrium	No new deficit; transient postoperative agitation/delirium (24–72 h)	Postop delirium in elderly; no tractography; ENDOPATH retractor
8	Left trigone/atrium	Transient aphasia + mild right arm weakness, resolved within 1–3 months	Association fibers peri-corridor edema; tractography performed; NICO retractor
11	Foramen of Monro	Permanent deficit due to suspected venous infarction (right-sided hemiplegia)	Deep venous injury (thalamostriate/septal/internal cerebral veins); tractography performed; ENDOPATH retractor

## Data Availability

The original data presented in this study are available on reasonable request from the corresponding author. The data are not publicly available due to privacy concerns.

## Abbreviations

The following abbreviations are used in this manuscript:

WHO	World Health Organization grading system
Fig.	figure
MRI	Magnetic Resonance Imaging
CT	computed tomography
Tab.	table
d	days
Y	years
M	male
F	female
P	patient
A	age
GTR	gross total removal
L	localization
L	left
R	right
PTV	preoperative tumor volume
D	tumor depth
EOR	extent of resection
NB	neurological status before surgery
LOC	loss of consciousness
R	retractor used (1: Endopath, 2: NICO)
LOR	length of retractor
COR	corticectomy dimension
NA	neurological status after surgery
LH	length of hospital stay
FU	months of follow-up
LP	late postoperative complications.
